# Smart care facilities space for employees

**DOI:** 10.1192/j.eurpsy.2021.710

**Published:** 2021-08-13

**Authors:** E. Abbasian

**Affiliations:** Architecture And Environmental Design, Iran University of science and technology, tehran, Iran

**Keywords:** biophilia, mental health, work place and environment behavioral stress

## Abstract

**Introduction:**

Smart care in offices and industrials approach has the best way for the results of staying healthy in the transmission chain and this issue is not only a moral necessity but also it can be a successful plan where personal care centers start keeping the patients, employees, and experts of healthcare ward healthy by digital industries. In this project, the environment along with the individual`s body scan and accessing his /her biomarkers, the environment mechanism will be approached to the welfare level to disappear the disease, then change the air by antiseptic materials for air conditioning desirably.

**Objectives:**

Designing the self-care environment by accessing the smart elements decreases the pathogenic factors in the environment, by scanning the individual`s case and inquiring from health base, the features of the environmental elements will be optimized to normal situation.

**Methods:**

Content analysis of environmental components of space and categorizing of sensors. Determining the basic model for programming Designing the architectural environment in accordance with the standards set in the previous section, preparation of the model with thermal, biological, biochemical,.sensors.

**Results:**

Designing the self-care environment by accessing the smart elements decreases the pathogenic factors in the environment, by scanning the individual`s case and inquiring from health base, the features of the environmental elements will be optimized to normal situation.

**Conclusions:**

By a positive design in architectural changes in care units in the field of public spaces, offices and industrial parts, we can easily control individual behaviors in the face of pandemic diseases and decline their psychic side effects.

**Conflict of interest:**

Our work experience requires investment to run on a real scale

